# LESSONS LEARNED FROM A FIVE-YEAR STUDY ON PREVENTING SELF-NEGLECT AMONG AT-RISK HEALTH CARE PATIENTS IN TEXAS

**DOI:** 10.1093/geroni/igae098.0144

**Published:** 2024-12-31

**Authors:** Farida Ejaz, Courtney Reynolds

**Affiliations:** Benjamin Rose Institute on Aging, Cleveland, Ohio, United States; Benjamin Rose, Cleveland, Ohio, United States

## Abstract

This presentation focuses on lessons learned from a study on preventing self-neglect among healthcare patients that is currently featured as a Success on the Administration for Community Living’s Elder Justice webpage. Collaborators included researchers, a large healthcare system, and Adult Protective Services. We focused on preventing self-neglect by identifying older and disabled patients from 19 primary care clinics with risk factors for self-neglect such as depression and dementia. Clinics were randomized to intervention (patients received 4-months of case management) and control (usual care) conditions, and 477 randomly selected patients participated. The sample was predominantly Hispanic (68%) and low income (< $1,361). The lessons learned have important implications for practice, policy, and future research. For example, language mattered when recruiting study participants and the term ‘self-neglect’ needed to be softened. Despite paying $25-$35 for an interview, the response rate was approximately 30%, leading to timeline delays. Intervention group patients (285) who were interviewed at home had higher response rates than control patients (192) interviewed at the clinic/other settings. Thus, conducting home assessments for self-neglect and offering case management to at-risk patients was feasible. In general, at-risk patients were not self-neglecting but had a variety of needs (823 total needs) such as food insecurity and home modifications and needed service referrals (total 1,226 referrals). Needs were met 65% of the time. Barriers to service utilization included refusals. Discussion will focus on how the lessons learned can help research-practice collaborations prevent self-neglect among low-income, minority populations and prepare for challenges in the field.

